# Immune Checkpoint-Related Gene Polymorphisms Are Associated With Primary Immune Thrombocytopenia

**DOI:** 10.3389/fimmu.2020.615941

**Published:** 2021-01-05

**Authors:** Shuwen Wang, Xiaoyu Zhang, Shaoqiu Leng, Qirui Xu, Zi Sheng, Yanqi Zhang, Jie Yu, Qi Feng, Ming Hou, Jun Peng, Xiang Hu

**Affiliations:** ^1^ Department of Hematology, Qilu Hospital, Cheeloo College of Medicine, Shandong University, Jinan, China; ^2^ Shandong Provincial Key Laboratory of Immunohematology, Qilu Hospital, Cheeloo College of Medicine, Shandong University, Jinan, China; ^3^ Department of Hematology, Weihai Municipal Hospital, Weihai, China

**Keywords:** primary immune thrombocytopenia, immune checkpoint, single-nucleotide polymorphism, T cell, CD28

## Abstract

Cancer immunotherapy by immune checkpoint blockade has been effective in the treatment of certain tumors. However, the association between immune checkpoints and autoimmune diseases remains elusive and requires urgent investigation. Primary immune thrombocytopenia (ITP), characterized by reduced platelet count and a consequent increased risk of bleeding, is an autoimmune disorder with a hyper-activated T cell response. Here, we investigated the contribution of immune checkpoint-related single-nucleotide polymorphisms (SNPs), including CD28, ICOS, PD1, TNFSF4, DNAM1, TIM3, CTLA4, and LAG3 to the susceptibility and therapeutic effects of ITP. In this case-control study, 307 ITP patients and 295 age-matched healthy participants were recruited. We used the MassARRAY system for genotyping immune checkpoint-related SNPs. Our results revealed that rs1980422 in CD28 was associated with an increased risk of ITP after false discovery rate correction (codominant, CT *vs.* TT, OR = 1.788, 95% CI = 1.178–2.713, p = 0.006). In addition, CD28 expression at both the mRNA and protein levels was significantly higher in patients with CT than in those with the TT genotype (p = 0.028 and p = 0.001, respectively). Furthermore, the T allele of PD1 rs36084323 was a risk factor for ITP severity and the T allele of DNAM1 rs763361 for corticosteroid-resistance. In contrast, the T allele of LAG3 rs870849 was a protective factor for ITP severity, and the T allele of ICOS rs6726035 was protective against corticosteroid-resistance. The TT/CT genotypes of PD1 rs36084323 also showed an 8.889-fold increase in the risk of developing refractory ITP. This study indicates that immune checkpoint-related SNPs, especially CD28 rs1980422, may be genetic factors associated with the development and treatment of ITP patients. Our results shed new light on prognosis prediction, disease severity, and discovering new therapeutic targets.

## Introduction

Primary immune thrombocytopenia (ITP), one of the most common bleeding disorders, is characterized by reduced platelet count and an increased risk of bleeding (1, 2). ITP is an acquired autoimmune disease, in which platelets are opsonized by auto-antibodies and destroyed by phagocytic cells (3–6). ITP pathogenesis involves a hyper-activated T cell response, which is important for cell-mediated cytotoxicity and IgG production (7–10). Therefore, investigating T cell abnormalities in ITP patients may reveal the mechanism of pathogenesis and development of ITP.

Immune checkpoints, including co-stimulation and co-inhibition signal pathways, are among the central mechanisms that regulate T-cell mediated immune responses (11, 12). Co-stimulation and co-inhibition signals, also termed “second signals,” modify the “first signal” provided by the T cell receptor (TCR) and MHC recognition, and determine the outcome of adaptive T cell immunity synergistically (13–16). As the repertoire of the TCR is generated randomly, the TCR can recognize and eliminate numerous antigens, including self-antigens (17). Thus, co-stimulation and co-inhibition signals are pivotal for maintaining self-tolerance. Aberrant expression of costimulatory molecules and co-inhibitory molecules may promote the generation of self-reactive T cells or cause evasion of self-reactive T cells from central and peripheral tolerance, contributing to autoimmunity (18, 19).

The costimulatory molecules of T cells consist of CD28, inducible costimulatory (ICOS), TNF superfamily member 4 (TNFSF4), and DNAM1 (CD226), and the co-inhibitory molecules contain TIM3, cytotoxic T-lymphocyte associated protein 4 (CTLA4), programmed death-1 (PD1), and lymphocyte activating 3 (LAG3) (20–24). Among these, CD28 and CTLA4 represent the best-studied costimulatory pathways. CD28 and CTLA4 interact with two ligands (CD80 and CD86) on the surface of antigen-presenting cells (APCs), introducing a positive stimulatory and a negative inhibitory signal into T cells, respectively (25). CD28, constitutively expressed on the surface of T cells, is important for T cell survival, proliferation, and effector function. Alternatively, CTLA4, which is highly expressed after T cell activation, acts as a competitor of CD28 and induces a state of T cell unresponsiveness and anergy (26–28). PD1, a novel co-inhibitory member of the B7/CD28 family, is engaged by PD-L1 to inhibit T cell activation (29–31). The PD1 inhibitor has been used in the clinical treatment of cancer to cancel the limitation on T cell-mediated anti-cancer responses (32–34). Thus, co-stimulation and co-inhibition signals may contribute to the hyper-active state of T cells in autoimmune diseases.

Single-nucleotide polymorphisms (SNPs) are the most common type of genetic variation among humans. Genetic studies have revealed that multiple polymorphisms in the genes encoding immune checkpoint molecules are associated with susceptibility to several autoimmune diseases. The CD28 rs1980422 CC genotype is associated with both rheumatoid factor (RF) and anti-citrullinated protein antibodies (ACPA) in rheumatoid arthritis (RA) (35, 36), while polymorphisms in ICOS rs6726035, PD1 rs36084323, DNAM1 rs763361, and TIM3 rs10515746 also act as related factors in RA development (37–40). The TT genotype of rs231779 in the CTLA4 gene increases one’s risk of Graves’ disease (41). In addition, there was a different distribution of the TT genotype in LAG3 rs870849 in multiple sclerosis (MS) patients compared to healthy controls (42). In systemic lupus erythematosus (SLE), the frequency of minor T alleles of TNFSF4 rs2205960 is associated with autoantibody production and is important in Chinese and Indian patients (43). As these SNPs are related to autoimmune diseases, few studies have focused on SNPs of immune checkpoint genes in ITP. Whether immune checkpoint gene polymorphisms are protective or risk factors in ITP and whether these SNPs are associated with susceptibility, severity, corticosteroid-sensitivity, or refractoriness of ITP are still largely unknown.

In this study, we hypothesized that these autoimmune disease-related SNPs in immune checkpoint genes may be associated with ITP. We explored the genetic variants in eight immune checkpoint genes using data from 602 genomes and performed a case-control association analysis with selected tagging SNPs in Chinese populations. Furthermore, CD28 expression was analyzed in ITP cases with different genotypes.

## Materials and Methods

### Patients and Controls

We recruited 307 patients with primary ITP between May 2016 and May 2020 from the Department of Hematology, Qilu Hospital, Cheeloo College of Medicine, Shandong University.

The diagnostic criteria were consistent with the International ITP guidelines (44). Specifically, the clinical diagnostic details included: (1) platelet count of peripheral blood <100 × 10^9^/L on at least two consecutive routine blood tests, (2) normal or increased megakaryocyte count in bone marrow, and (3) no other diseases or conditions related to thrombocytopenia. Inclusion in the study required the absence of other causes of secondary thrombocytopenia based on patient history, physical examination, clinical manifestations, and laboratory tests. Patients with other autoimmune or hemorrhagic diseases (*e.g.*, SLE, severe anemia), or thrombocytopenia due to pregnancy, viruses (*e.g.*, hepatitis C virus, human immunodeficiency virus), active infections, vaccinations, or drugs (*e.g.*, heparin) were not included in this study.

Based on disease progression and treatment response, patients were further stratified by the following three indicators: severity, refractoriness, and corticosteroid sensitivity. Severe ITP describes patients with a platelet count <10 × 10^9^/L and active bleeding, or with bleeding symptoms sufficient to require treatment or additional intervention, such as a dose increase or the use of another platelet-enhancing drug to relieve thrombocytopenia. Refractory ITP describes patients who have had a splenectomy that was not effective or with postoperative recurrence, and patients with severe ITP or bleeding trends that require intervention. Corticosteroid therapy is a first-line treatment for ITP, including high-dose dexamethasone 40 mg/d for 4 d (patients who did not respond could repeat one cycle within two weeks) or prednisone 1.0 mg/kg/d for 4 weeks. Corticosteroid-sensitive described patients with a platelet count no less than 30 × 10^9^/L with at least a 2-fold increase compared with the baseline count and no bleeding after intervention. Patients were considered corticosteroid-resistant if they required additional treatment.

We recruited 295 age-matched healthy participants as the control group. We randomly recruited healthy participants from a group of healthy volunteers with no active infection or other autoimmune diseases, and no symptoms of thrombocytopenia. In this study, all patients and controls were Chinese, and no genetic associations were found among participants.

The Medical Ethics Committee of Qilu Hospital, Cheeloo College of Medicine, Shandong University reviewed and approved this study. Informed consent was obtained from all patients and controls in accordance with the Declaration of Helsinki.

### DNA Extraction and Genotyping

Peripheral venous blood was collected from all participants (5 ml) and peripheral blood mononuclear cells (PBMCs) were isolated by Ficoll density gradient centrifugation. Genomic DNA was extracted from PBMCs using a commercial DNA extraction kit (TianGen, China). We used a spectrophotometer to detect the concentration and purity of the DNA extraction. Before genotyping, we stored extracted DNA at −80°C. We selected SNPs associated with T-cell immune checkpoints (summarized in **Table 1**). The time-of-flight mass spectrometry (MassARRAY) system (BGI Tech., China) was used to detect the genotype of selected SNPs. We performed genotyping *via* PCR amplification, shrimp alkaline phosphatase enzyme treatment, single base extension, resin desalination purification, and mass spectrometry.

**Table 1:** Selected genes and SNPs.

Genes	SNPs
TIM3	rs10515746
CD28	rs1980422
TNFSF4	rs2205960
CTLA4	rs231779
PD1	rs36084323
ICOS	rs6726035
DNAM1	rs763361
LAG3	rs870849

SNP, single-nucleotide polymorphism.

### Flow Cytometry

The phenotypes of PBMCs were analyzed for cell surface markers CD3, CD4, and CD28. Cells were incubated with anti-CD3, anti-CD4, and anti-CD28 mAbs (Biolegend, USA; 30 min, 4°C, dark). Data were acquired using a Gallios Flow Cytometer (Beckman Coulter Inc., USA). A total of 200,000 events per tube were analyzed using Kaluza Flow Cytometry Analysis Software (Beckman Coulter Inc.).

### RNA Extraction and Real Time RT-PCR

CD4^+^ T cells were separated from PBMCs of patients who were admitted to the hospital without receiving any ITP-specific treatments (corticosteroids, IVIG, rituximab, and TPO-RA) within 3 months, using a CD4^+^ T cell isolation kit (Miltenyi Biotec, Germany). The purity of CD4^+^ cells was >98% according to flow cytometry. Total RNA was extracted from CD4^+^ T cells using TRIzol reagent (Invitrogen Life Technologies, USA), and RNA was converted into cDNA using the PrimeScript RT Reagent Kit Perfect Real Time (Takara Bio, Japan). Quantitative PCR was performed on the LightCycler 480II Real-Time PCR system (Roche, Switzerland) according to the standard protocol. The primers used are listed below.

- CD28 forward: CTATTTCCCGGACCTTCTAAGCC
- CD28 reverse: GCGGGGAGTCATGTTCATGTA
- GAPDH forward: GCTCTCTGCTCCTCCTGTT
- GAPDH reverse: GTTGACTCCGACCTTCACCT

Quantitative PCR included a 20 μl volume composed of 0.5 μl of the forward and reverse primers, 5 μl cDNA, 4.5 μl ddH_2_O, and 10 μl SYBR Green Real-time PCR Master Mix. We normalized the expression of target genes (CD28) to that of the internal standard gene (GAPDH), and mRNA expression was analyzed using the 2^−ΔΔCt^ method.

### Statistical Analysis

We used the calculator on the Helmholtz Centre website in Munich to calculate the p value of the Hardy Weinberg equilibrium. We analyzed genotyping data using four models, including the dominant, recessive, codominant, and allelic frequency models. For preliminary screening, we used Fisher’s exact test or the chi-squared (χ^2^) test to analyze the relationships between SNPs and the susceptibility, severity, refractoriness, and corticosteroid sensitivity of ITP. Odds ratios (ORs) and adjusted p-values were analyzed by univariate and multivariate binary logistic regression analyses with 95% confidence intervals (95% CIs). SPSS 26.0 statistical software (SPSS, Inc., USA) was used for statistical analyses. In addition, generalized multifactor dimensionality reduction (GMDR) was performed to detect gene-gene interactions with GMDR 0.9 software. Statistically significant differences were defined as those with a p-value <0.05 or a false discovery rate (FDR) q value <0.05.

## Results

### Study Population

Participant details are shown in **Table 2**, including demographic and clinical characteristics. All eight SNPs were in accordance with Hardy Weinberg equilibrium in the control group (p > 0.24, **Supplementary Table 1**).

**Table 2:** Demographic and clinical characteristics.

	ITP patients	Controls
No.	307	295
Age, mean ± SD	44.92 ± 14.02	47.61 ± 16.93
Sex (M/F)	117/190	134/161
ITP severity, *n* (%)		
Severe ITP	137 (44.6)	N/A
Non-severe ITP	170 (55.4)	N/A
Treatment, *n* (%)		
No use of corticosteroid	63 (20.5)	N/A
Corticosteroid-sensitive	107 (34.9)	N/A
Corticosteroid-resistant	137 (44.6)	N/A
Refractory ITP	27 (8.8)	N/A
Non-refractory ITP	280 (91.2)	N/A

F, female; ITP, immune thrombocytopenia; M, male; N/A, not applicable.

### Polymorphisms Associated With Immune Thrombocytopenia Susceptibility

We used four genetic models to analyze the relationship between eight immune checkpoint-related SNPs and ITP susceptibility. We performed preliminary screening to analyze the association between each SNP and ITP susceptibility. The data are detailed in **Supplementary Table 1**. Under codominant, dominant, and allele models, allelic and genotypic frequencies of TIM3 rs10515746 were significantly related to ITP susceptibility (p < 0.05). The different distributions of rs1980422 polymorphism in CD28 under codominant and dominant models showed a relationship with ITP susceptibility (p < 0.05), which was also related to ITP susceptibility after FDR correction. In addition, the allele and recessive models indicated an association between ICOS rs6726035 and ITP susceptibility (p < 0.05).

The CA genotype and allele A in place of C of rs10515746 in TIM3 was significantly related to ITP susceptibility after adjusting for sex and age (p = 0.027 and p = 0.029, respectively; **Table 3**). Regarding rs1980422 in CD28, under codominant and dominant models, the CT and CC/CT genotypes were associated with ITP susceptibility (p = 0.006 and p = 0.016, respectively; **Table 3**). Moreover, the T allele and TT genotype of ICOS rs6726035 showed a significant association with ITP susceptibility (p = 0.023 and p = 0.032, respectively; **Table 3**). Notably, all three of these polymorphisms revealed an increased susceptibility to ITP (OR > 1, p < 0.05, **Table 3**). However, after FDR correction, only CD28 rs1980422 was significantly associated with ITP susceptibility in univariate logistic regression analysis.

**Table 3:** Selected SNPs associated with ITP risk.

Gene	SNP	Model	Genotype/allele	Control		ITP		OR (95% CI)	Adjusted *p* value
				Count	%	Count	%		
TIM3	rs10515746	Codominant	CC	291	98.6	293	95.4	1.000	
			AA	0	0.0	0	0.0	_	_
			CA	4	1.4	14	4.6	3.572 (1.153–11.064)	**0.027**
		Dominant	CC	291	98.6	293	95.4	1.000	
			CA/AA	4	1.4	14	4.6	3.628 (1.175–11.200)	**0.025**
		Allele	C	586	99.3	600	97.7	1.000	
			A	4	0.7	14	2.3	3.509 (1.140–10.798)	**0.029**
CD28	rs1980422	Codominant	TT	246	83.4	233	75.9	1.000	
			CC	4	1.4	0	0.0	_	0.999
			CT	45	15.2	74	24.1	1.798 (1.181–2.711)	**0.006**
		Dominant	TT	246	83.4	233	75.9	1.000	
			CC/CT	49	16.6	74	24.1	1.646 (1.095–2.472)	**0.016**
ICOS	rs6726035	Recessive	TT	62	21.0	89	29.0	1.000	
			CC/CT	233	79.0	218	71.0	1.510 (1.037–2.198)	**0.032**
		Allele	C	320	54.2	292	47.6	1.000	
			T	270	45.8	322	52.4	1.303 (1.037–1.637)	**0.023**

CI, confidence interval; ITP, immune thrombocytopenia; OR, odds ratio; SNP, single-nucleotide polymorphism. Adjusted p value calculated with univariate logistic regression. Bold highlights p < 0.05.

Thereafter, combined analysis under the codominant model by multivariate logistic regression analysis revealed that the heterozygous genotypes of TIM3 rs10515746 and CD28 rs1980422 played a significant role in increased risk of ITP (OR = 3.509, 95% CI = 1.128–10.914, p = 0.03; OR = 1.788, 95% CI = 1.178–2.713, p = 0.006, respectively, **Table 4**). In addition, analysis using the dominant model showed that the CA/AA genotypes of TIM3 rs10515746 and CC/CT genotypes of CD28 rs1980422 significantly increased the ITP risk compared with homozygous major alleles (OR = 3.617, 95% CI = 1.170–11.185, p = 0.026 and OR = 1.645, 95% CI = 1.093–2.475, p = 0.017, respectively, **Table 4**). The allelic distribution of TIM3 rs10515746 and ICOS rs6726035 was also related to ITP susceptibility after combined analysis (OR = 3.218, 95% CI = 1.043–9.924, p = 0.042 and OR = 1.276, 95% CI = 1.015–1.605, p = 0.037, respectively; **Table 4**).

**Table 4:** Selected SNPs associated with ITP risk by multivariate logistic regression analysis.

Gene	SNP	Model	Genotype/allele	Controls		ITP patients		OR (95% CI)	Adjusted *p* value
				Count	%	Count	%		
TIM3	rs10515746	Codominant	CC	291	98.6	293	95.4	1.000	
			AA	0	0.0	0	0.0	_	_
			CA	4	1.4	14	4.6	3.509 (1.128–10.914)	**0.030**
		Dominant	CC	291	98.6	293	95.4	1.000	
			CA/AA	4	1.4	14	4.6	3.617 (1.170–11.185)	**0.026**
		Allele	C	586	99.3	600	97.7	1.000	
			A	4	0.7	14	2.3	3.218 (1.043–9.924)	**0.042**
CD28	rs1980422	Codominant	TT	246	83.4	233	75.9	1.000	
			CC	4	1.4	0	0.0	_	0.999
			CT	45	15.2	74	24.1	1.788 (1.178–2.713)	**0.006**
		Dominant	TT	246	83.4	233	75.9	1.000	
			CC/CT	49	16.6	74	24.1	1.645 (1.093–2.475)	**0.017**
ICOS	rs6726035	Allele	C	320	54.2	292	47.6	1.000	
			T	270	45.8	322	52.4	1.276 (1.015–1.605)	**0.037**

CI, confidence interval; ITP, immune thrombocytopenia; OR, odds ratio; SNP, single-nucleotide polymorphism. Adjusted p value calculated with multivariate logistic regression. Bold highlights p < 0.05.

**Figure 1:**
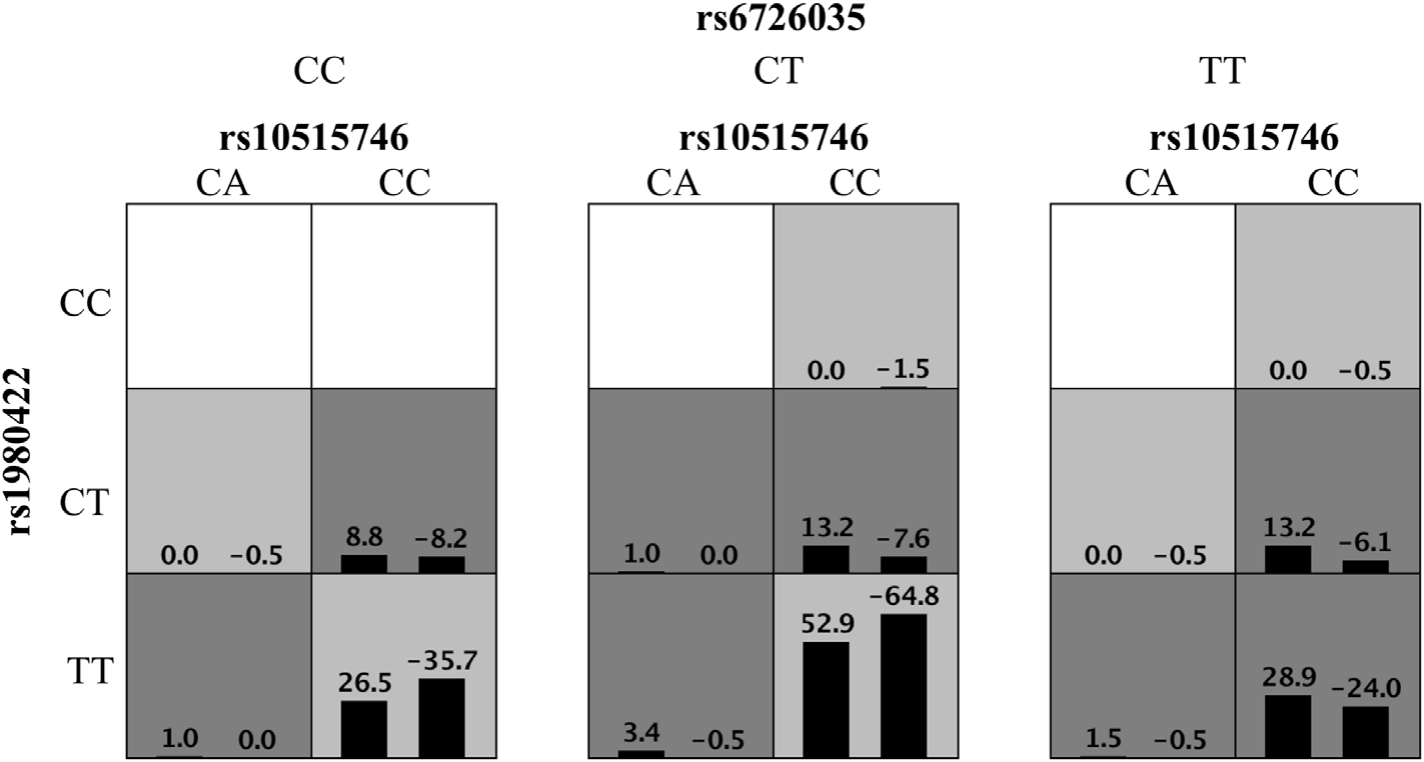
Distribution of high-risk genotypes and protective genotypes in the GMDR model. High risk genotypes (dark gray) and protective genotypes (light gray) combinations are presented. Patterns of high-risk genotypes and protective genotypes differ across different multi-locus dimensions; this is evidence of epistasis or gene–gene interaction.

High-order interactions were further investigated for ITP susceptibility using the GMDR method. Based on the above results, TIM3 rs10515746, CD28 rs1980422, and ICOS rs6726035 were included as variables in GMDR analysis. The data showed that the three-locus model was the optimal model, with the best cross-validation consistencies of 10/10 and p = 0.001 (**Figure 1**). These results indicated that these three SNPs exhibited interactive effects on ITP susceptibility.

### Polymorphisms Associated With Immune Thrombocytopenia Severity

We divided ITP patients into non-severe and severe groups to examine the association between selected immune checkpoint-related SNPs and ITP severity. Allelic and genotypic frequencies of PD1 rs36084323 under the four models and LAG3 rs870849 under the allele model were statistically different between non-severe and severe groups (p < 0.05, **Supplementary Table 2**).

Following adjustment for age and sex, the TT genotype and T allele of PD1 rs36084323 were significantly associated with ITP severity in the four models (p = 0.008, p = 0.029, p = 0.039 and p = 0.012, respectively; **Table 5**). Under the allele model, the T allele of LAG3 rs870849 showed a statistical relationship with ITP severity (p = 0.022, **Table 5**). Combined analysis with the allele model revealed that the minor alleles of PD1 rs36084323 and LAG3 rs870849 play different roles regarding ITP severity. Specifically, ITP patients carrying the T allele of PD1 rs36084323 showed a 1.649-fold increased risk of developing severe ITP (OR = 1.649, 95% CI = 1.186–2.291, p = 0.003, **Table 5**). In contrast, patients with the T allele of LAG3 rs870849 showed a 0.506-fold decreased risk of severe ITP (OR = 0.506, 95% CI = 0.312–0.818, p = 0.005, **Table 5**).

**Table 5:** Selected SNPs associated with ITP severity.

Gene, SNP	Model	Genotype/allele	Non-severe		Severe		OR (95% CI)^#^	Adjusted *p* value^#^	OR (95% CI)*	Adjusted *p* value*
			Count	%	Count	%				
PD1, rs36084323	Codominant	CC	48	28.2	24	17.5	1.000			
		TT	28	16.5	36	26.3	2.558 (1.274–5.138)	**0.008**		
		CT	94	55.3	77	56.2	1.640 (0.922–2.919)	0.093		
	Dominant	CC	48	28.2	24	17.5	1.000			
		TT/CT	122	71.8	113	82.5	1.851 (1.064–3.221)	**0.029**		
	Recessive	TT	28	16.5	36	26.3	1.000			
		CC/CT	142	83.5	101	73.7	1.797 (1.029–3.138)	**0.039**		
	Allele	C	190	55.9	125	45.6	1.000		1.000	
		T	150	44.1	149	54.4	1.507 (1.093–2.076)	**0.012**	1.649 (1.186–2.291)	**0.003**
LAG3, rs870849	Allele	C	280	82.4	243	88.7	1.000		1.000	
		T	60	17.6	31	11.3	0.579 (0.362–0.925)	**0.022**	0.506 (0.312–0.818)	**0.005**

CI, confidence interval; ITP, immune thrombocytopenia; OR, odds ratio; SNP, single-nucleotide polymorphism. Calculated with ^#^univariate or *multivariate logistic regression under allele model. Bold highlights p < 0.05.

### Polymorphisms Associated With Corticosteroid Sensitivity

Thereafter, we explored the association between immune checkpoint-related SNPs and response to ITP treatment, especially corticosteroid therapy. Similarly, we used four models to study corticosteroid sensitivity and resistance among ITP patients. The patients who were administered corticosteroid therapy were stratified into the corticosteroid-sensitive group and corticosteroid-resistant group (n = 107 and n = 137, respectively).

Statistical analysis under the four models revealed a different distribution of genotypic and allelic frequencies of DNAM1 rs763361, indicating that it was significantly associated with corticosteroid-sensitivity (p < 0.05, **Supplementary Table 3**). ICOS rs6726035 under the allele model was also related to corticosteroid-sensitivity of ITP patients (p < 0.05, **Supplementary Table 3**).

For DNAM1 rs763361, after adjusting for sex and age, minor allele homozygotes rather than heterozygotes were significantly associated with corticosteroid-sensitivity in the codominant and recessive models (p = 0.016 and p = 0.030, respectively, **Table 6**). Allelic frequencies of ICOS rs6726035 were significantly different under the allele model between corticosteroid-sensitive and -resistant groups (p = 0.015, **Table 6**).

**Table 6:** Association between selected SNPs and corticosteroid-sensitivity of ITP patients.

Gene, SNP	Model	Genotype/allele	Corticosteroid-sensitive		Corticosteroid-resistant		OR (95% CI)^#^	Adjusted *p* value^#^	OR (95% CI)*	Adjusted *p* value*
			Count	%	Count	%				
DNAM1, rs763361	Codominant	CC	56	52.3	54	39.4	1.000			
		TT	5	4.7	18	13.1	3.719 (1.276–10.838)	**0.016**		
		TC	46	43.0	65	47.5	1.413 (0.825–2.421)	0.208		
	Recessive	TT	5	4.7	18	13.1	1.000			
		CC/TC	102	95.3	119	86.9	3.151 (1.116–8.892)	**0.030**		
	Allele	C	158	73.8	173	63.1	1.000		1.000	
		T	56	26.2	101	36.9	1.623 (1.092–2.412)	**0.017**	1.939 (1.278–2.942)	**0.002**
ICOS, rs6726035	Allele	C	89	41.6	143	52.2	1.000		1.000	
		T	125	58.4	131	47.8	0.634 (0.439–0.914)	**0.015**	0.538 (0.366–0.791)	**0.002**

CI, confidence interval; ITP, immune thrombocytopenia; OR, odds ratio; SNP, single-nucleotide polymorphism. Calculated with **^#^**univariate or *multivariate logistic regression under allele model.

Bold highlights p < 0.05.

Results under the allele model revealed statistical associations between DNAM1 rs763361, ICOS rs6726035, and corticosteroid sensitivity determined using multivariate logistic regression analysis (p = 0.002 and p = 0.002, respectively; **Table 6**). The T allele of DNAM1 rs763361 was associated with a 1.939-fold increased risk of corticosteroid-resistance (OR = 1.939, 95% CI = 1.278–2.942, p = 0.002, **Table 6**). Conversely, the T allele of ICOS rs6726035 had a protective effect (OR = 0.538, 95% CI = 0.366–0.791, p = 0.002, **Table 6**).

### Polymorphisms Associated With Immune Thrombocytopenia Refractoriness

ITP patients were further divided into refractory and non-refractory groups to study the influence of polymorphisms on ITP refractoriness. We compared the distribution of alleles and genotypes between both groups using Fisher’s exact test or the χ^2^ test. The genotypic distribution of PD1 rs36084323 was significantly associated with ITP refractoriness (p < 0.05, **Supplementary Table 4**).

Following adjustment for age and sex, the genotypic distribution of PD1 rs36084323 under the codominant and dominant models was statistically different between both groups (p = 0.030 and p = 0.034, respectively; **Table 7**). In addition, the TT/CT genotypes of rs36084323 showed an 8.889-fold increased risk of developing refractory ITP compared to the CC genotype under the dominant model (OR = 8.889, 95% CI = 1.183–66.771, p = 0.034, **Table 7**).

**Table 7:** Selected SNPs associated with ITP refractoriness.

Gene, SNP	Model	Genotype	Non-refractory ITP		Refractory ITP		OR (95% CI)	Adjusted *p* value
			Count	%	Count	%		
PD1, rs36084323	Codominant	CC	71	25.4	1	3.7	1.000	
		TT	58	20.7	6	22.2	7.314 (0.854–62.612)	0.069
		CT	151	53.9	20	74.1	9.500 (1.249-72.289)	**0.030**
	Dominant	CC	71	25.4	1	3.7	1.000	
		TT/CT	209	74.6	26	96.3	8.889 (1.183–66.771)	**0.034**

CI, confidence interval; ITP, immune thrombocytopenia; OR, odds ratio; SNP, single-nucleotide polymorphism.

Adjusted p value calculated with univariate logistic regression. Bold highlights p < 0.05.

### CD28 rs1980422 Polymorphism Associated With CD28 Expression

The pathogenesis and development of ITP involves a hyper-activated T cell response, which includes the production of immunological effector proteins (45). To explore the relationship between the gene polymorphism and CD28 expression, we examined CD28 gene expression at the mRNA level by RT-PCR and at the protein level by flow cytometry. This was done considering that flow cytometry could be used to show the expression of the CD28 protein on the cell membrane of living CD4^+^ T cells, and real time PCR is the gold standard for detecting mRNA expression and is widely used in multiple studies.

**Figure 2:**
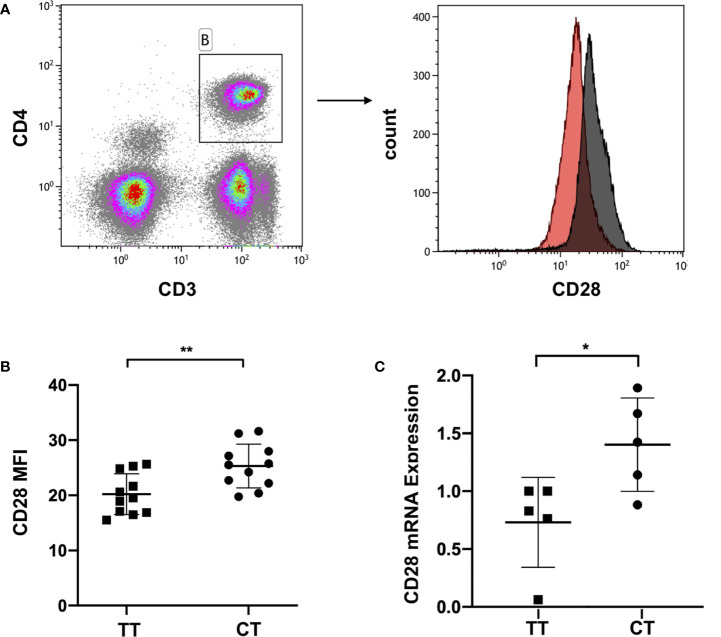
CD28 expression in ITP patients with TT or CT genotypes. **(A)** Representative plot of CD28 expression within the gate of CD4^+^ T cells. **(B)** CD28 expression at the protein level, and **(C)** mRNA level of ITP patients with the TT (n = 11 and n = 5, respectively) or CT genotypes (n = 11 and n = 5, respectively). **p < 0.01; *p < 0.05.

We collected peripheral blood of ITP patients with the CT genotype or TT genotype of CD28 rs1980422. According to the analysis of the mean fluorescence intensity (MFI) of CD28, ITP patients with the CT genotype showed a higher level of CD28 protein expression than patients with the TT genotype (p = 0.006, **Figures 2A, B**), although the percentages of CD28^+^ cells in CD4^+^ T cells were all greater than 83% and showed no significant difference between the two groups (p > 0.05, **Supplementary Figure 1**). The MFI of CD28 and the percentages of CD28^+^ cells in CD4^+^ T cells are detailed in **Supplementary Figure 2**. The CT genotype was also associated with increased CD28 mRNA levels compared to the TT genotype (p = 0.028, **Figure 2C**). The results indicate that the CT genotype of CD28 rs1980422 is a risk factor for ITP.

## Discussion

In this study, we examined the associations between CD28, CTLA4, DNAM1, ICOS, LAG3, PD1, TIM3, and TNFSF4 gene polymorphisms and ITP. We observed increased frequencies of heterozygous minor genotypes of TIM3 rs10515746, CD28 rs1980422, and the homozygous minor allele of ICOS rs6726035 in ITP patients compared with healthy controls, while only CD28 rs1980422 remained significant under the codominant model after FDR correction (p = 0.002). Moreover, the combination of TIM3 rs10515746, CD28 rs1980422, and ICOS rs6726035 best predicted a high risk of ITP. In addition, the T allele of PD1 rs36084323 corresponded to an increased risk of developing severe ITP, while the T allele of LAG3 rs870849 corresponded to a decreased risk of severe ITP. The PD1 rs36084323 polymorphism was also a risk factor for ITP refractoriness under the dominant and codominant models. Regarding corticosteroid sensitivity, individuals carrying TT genotypes of DNAM1 rs763361 had an increased risk of corticosteroid resistance in ITP. However, neither allelic nor genotypic frequencies of CTLA4 rs231779 and TNFSF4 rs2205960 were significantly associated with the susceptibility, severity, refractoriness, or corticosteroid sensitivity of ITP (p > 0.05, **Supplementary Tables 1–4**). Our results suggest that checkpoint molecules may be involved in the pathogenesis and development of ITP, and provide potential immune indicators for clinical treatment.

In autoimmunity, the implications of immune checkpoint molecules have been demonstrated in several autoimmune diseases, including SLE, RA, MS, and type 1 diabetes. CD28 blockade or deficiency delays and diminishes symptoms in an SLE mouse model (26, 46). The B7-CD28 costimulatory signal also promotes priming of auto-reactive T cells during the development of experimental autoimmune encephalomyelitis (EAE) (19). In contrast, blockade of CTLA4 accelerates EAE development, and PD1 deficiency in mice triggers a lupus-like disease (47, 48). In ITP, PD1 expression increased in CD4^+^ T cells and CD8^+^ T cells, while PD-L1 expression on monocyte-derived DCs was lower in patients with active ITP than in healthy controls (49, 50). A single high-dose dexamethasone treatment limited CD28 expression and enhanced CTLA4 expression in ITP patients (51). These studies have revealed that immune checkpoints may contribute to the immunopathogenesis of ITP. Our research investigated multiple autoimmune disease-related SNPs in checkpoint molecules and found that CD28 rs1980422 is a risk factor for ITP susceptibility, which provides new evidence of the impact of immune checkpoints on ITP.

The CD28 rs1980422 polymorphism has been investigated in multiple autoimmune diseases. These studies found that the minor allele of CD28 rs1980422 was not associated with lupus susceptibility in the Egyptian population or RA susceptibility in the Polish population (52, 53). However, association results in the Egyptian population revealed a strong association between CD28 and RA at both the genotypic and allelic levels (54). Our results suggest that individuals with the CT genotype on the CD28 rs1980422 locus showed high levels of susceptibility to ITP in the Chinese population, which confers the importance of CD28 rs1980422 in susceptibility to autoimmune diseases.

Several studies have focused on the association between genetic polymorphisms and ITP. In the Egyptian population, the T-G haplotype of the CD40 gene SNP rs1883832 is associated with an increased risk of ITP development (55), which also suggested that the immune checkpoint pathway may be involved in the pathogenesis and development of ITP. In addition, a case-control association analysis of 277 Chinese children revealed that abnormal expression, instead of genetic polymorphisms of CTLA4, may be correlated with susceptibility to ITP (56). Here, we found that mRNA and protein levels of CD28 in the CT genotype were higher than those in TT genotype in T cells of ITP patients, suggesting that heterozygotes of CD28 rs1980422 play an important role in increased expression of CD28 in ITP. Given that the rs1980422 SNP is located approximately 10 kb away from the 3′ untranslated region of CD28 and approximately 100 kb away from the 5′ untranslated region of CTLA4 (46), and the intergenetic sequence has been reported to regulate the transcription level of the genes nearby (57–59), it is also possible that the rs1980422 SNP is involved in the balance between CD28 and CTLA4 expression.

We have previously reported that TNFAIP3 rs10499194 and CARD9 rs4077515 are important susceptibility-related SNPs for ITP (60, 61). TNFAIP3 is important for the survival of CD4^+^ T cells, and downregulated expression of TNFAIP3 may contribute to T cell dysfunction in SLE (62, 63). CARD9 is important for the activation of dendritic cells, mediating naïve T cells to IL-17 producing Th17 cells (64). These results suggest the core role of dysregulated T cell function in ITP.

In summary, we have found that immune checkpoint-related SNPs, especially CD28 rs1980422, may be genetic factors associated with the development and treatment of ITP. Our results provide new clues for the identification of therapeutic targets and prognosis prediction in ITP.

## Data Availability Statement

The raw data supporting the conclusions of this article will be made available by the authors, without undue reservation.

## Ethics Statement

The studies involving human participants were reviewed and approved by The Medical Ethics Committee of Qilu Hospital, Cheeloo College of Medicine, Shandong University. Written informed consent to participate in this study was provided by the participants’ legal guardian/next of kin. Written informed consent was obtained from the individuals, and minors’ legal guardian/next of kin, for the publication of any potentially identifiable images or data included in this article.

## Author Contributions

XH and JP designed the research, analyzed the data, and wrote the paper. SW performed the research, analyzed the data, and wrote the paper. XZ, SL, QX, ZS, YZ, JY, and QF performed the research and evaluated the data. MH designed the research and reviewed the work, and all authors read and edited the manuscript. All authors contributed to the article and approved the submitted version.

## Funding

This work was supported by grants from the National Natural Science Foundation of China (91942306, 81770133, 81900122, and 81800112), China Postdoctoral Science Foundation (2020M672075), and the Shandong Provincial Key Research and Development Program (2019JZZY011016).

## Conflict of Interest

The authors declare that the research was conducted in the absence of any commercial or financial relationships that could be construed as a potential conflict of interest.
