# Supplementary material for: Immune Checkpoint-Related Gene Polymorphisms Are Associated With Primary Immune Thrombocytopenia
Source: Front Immunol. 2021 Jan 5;11:615941. doi: 10.3389/fimmu.2020.615941 (PMC7874092; doi:10.3389/fimmu.2020.615941)
Supplement: Supplementary file 1 [file DataSheet_1.pdf]

Immune Checkpoint-Related Gene Polymorphisms Are Associated with Primary Immune Thrombocytopenia

Shuwen Wang[1], Xiaoyu Zhang[1], Shaoqiu Leng[1], Qirui Xu[1], Zi Sheng[1], Yanqi Zhang[2], Jie Yu[3], Qi Feng[1], Ming Hou[1], Jun Peng[1*], Xiang Hu[2*]

Supplementary Figure 1

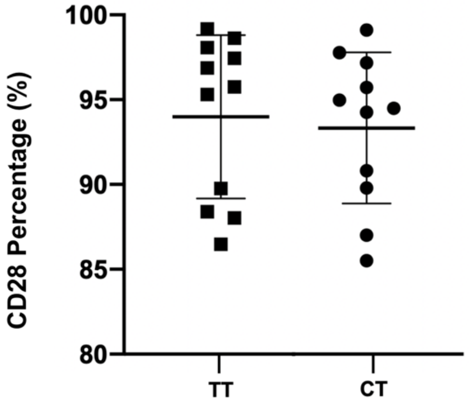

Supplementary Figure 1. CD28 percentages in ITP patients with TT or CT genotypes. Percentages of CD28+ cells in CD4+ T cells of ITP patients with the TT (n = 11) or CT (n = 11) genotypes. $p > 0.05$.

Supplemental Figure 2

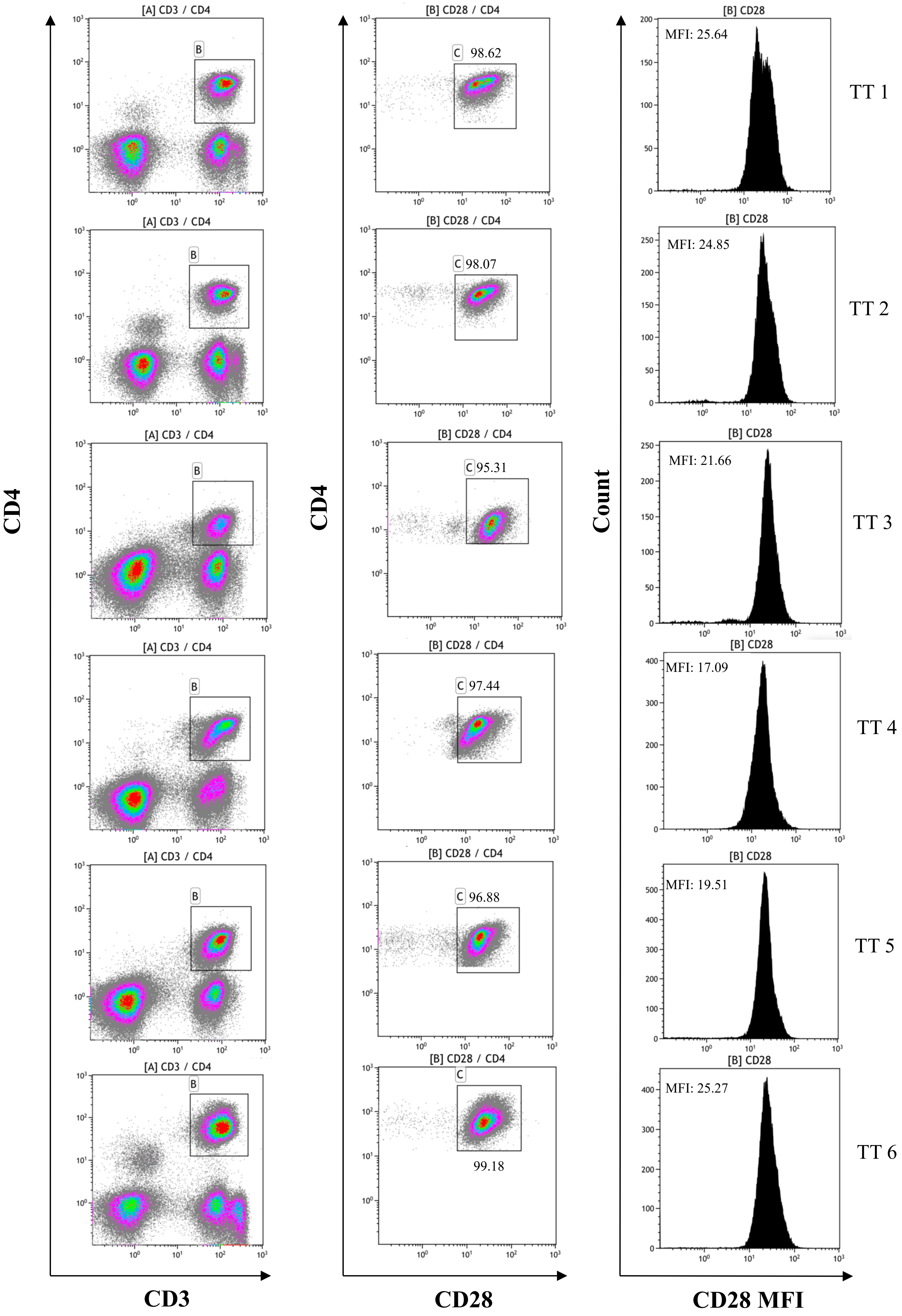

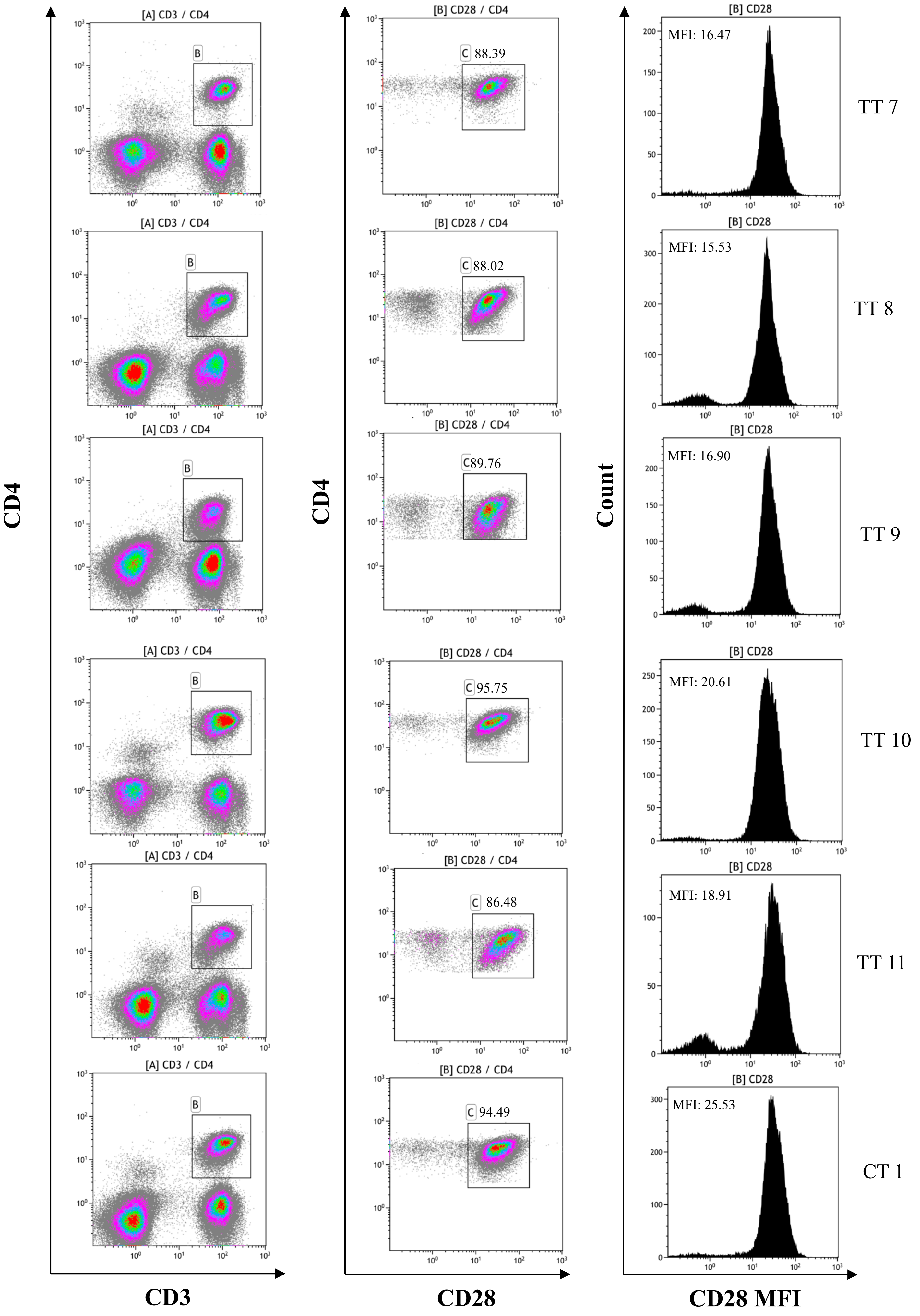

CD4
CD3
CD28
Count
CD28 MFI
[A] CD3 / CD4
[B] CD28 / CD4
[B] CD28
B
C 88.39
C 88.02
C89.76
C 95.75
C 86.48
C 94.49
MFI: 16.47
MFI: 15.53
MFI: 16.90
MFI: 20.61
MFI: 18.91
MFI: 25.53
TT 7
TT 8
TT 9
TT 10
TT 11
CT 1

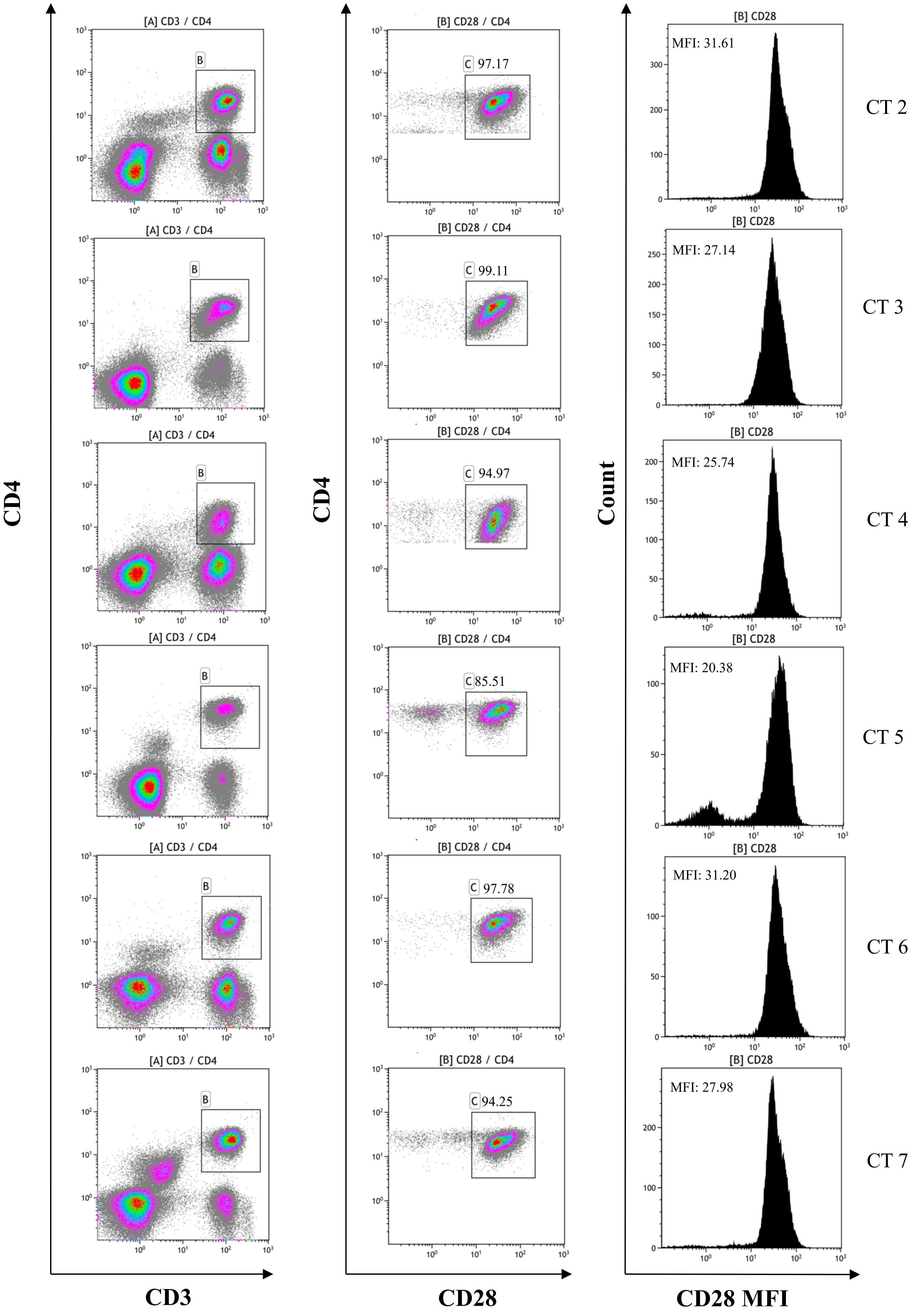

[A] CD3 / CD4
B
[B] CD28 / CD4
C 97.17
C 99.11
C 94.97
C 85.51
C 97.78
C 94.25
[B] CD28
MFI: 31.61
MFI: 27.14
MFI: 25.74
MFI: 20.38
MFI: 31.20
MFI: 27.98
CT 2
CT 3
CT 4
CT 5
CT 6
CT 7
CD4
CD3
CD28
Count
CD28 MFI

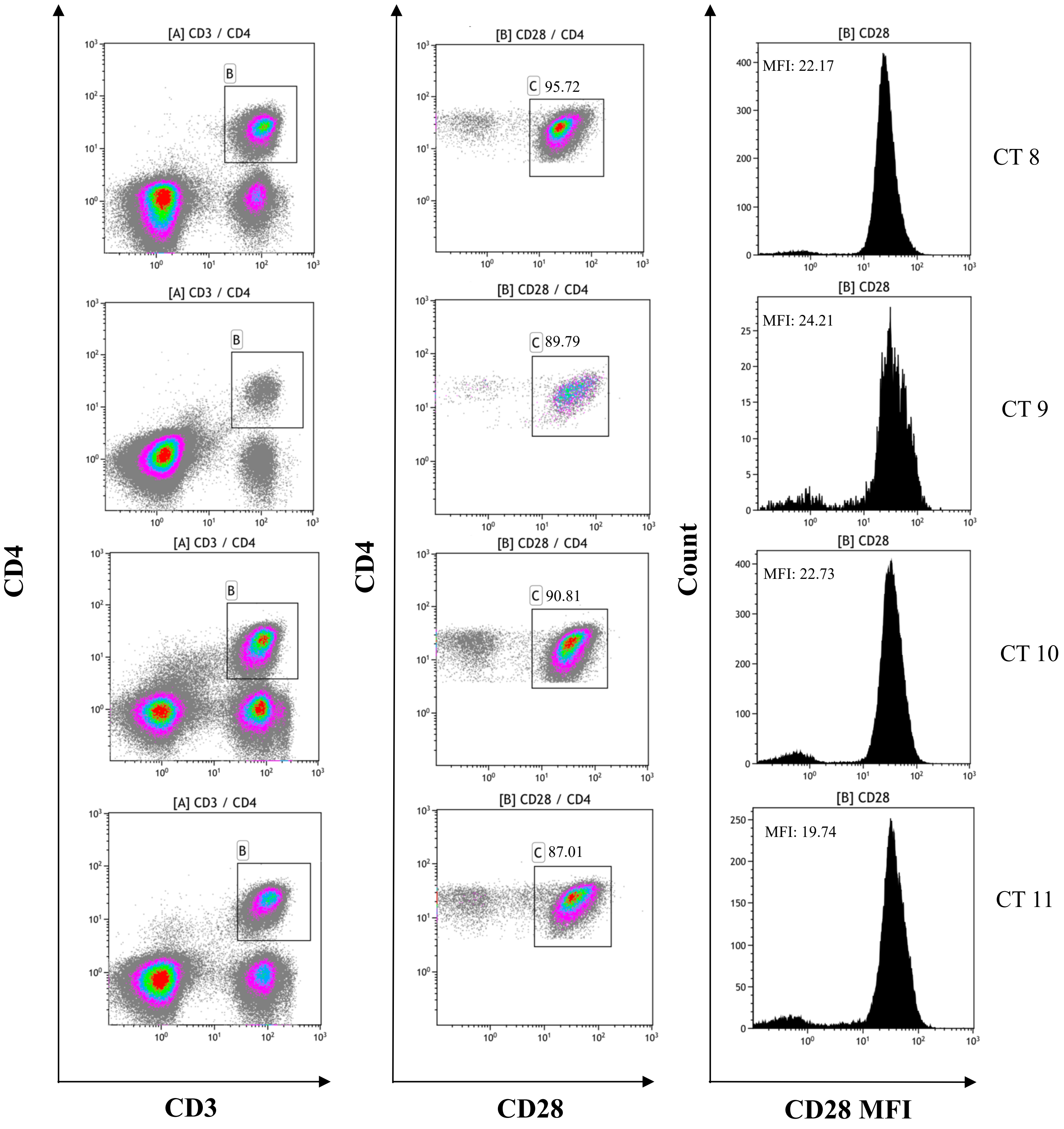

Supplementary Figure 2. Flow cytometric analysis of ITP patients with TT or CT genotypes. Gating the $CD3^{+}CD4^{+}$ cells of ITP patients with the TT (n = 11) or CT (n = 11) genotypes as gate B, then analyzing the percentages of $CD28^{+}$ cells and the mean fluorescence intensity of CD28 in gate B.

Supplementary Table S1

HWE test and association between SNPs and ITP susceptibility.

Genes	SNPs	Genotype	Allele	Controls		ITP		HWE -p	Model / allele	Uncorrected p value
				Count	%	Count	%			
TIM3	rs10515746	CC		291	98.6	293	95.4	0.907	Codominant	**0.021**
		AA		0	0.0	0	0.0		Dominant	**0.021**
		CA		4	1.4	14	4.6		Recessive	_
			C	586	99.3	600	97.7		Allele	**0.022**
			A	4	0.7	14	2.3			
CD28	rs1980422	TT		246	83.4	233	75.9	0.249	Codominant	**0.002**
		CC		4	1.4	0	0.0		Dominant	**0.023**
		CT		45	15.2	74	24.1		Recessive	0.057
			T	537	91.0	540	87.9		Allele	0.083
			C	53	9.0	74	12.1			
TNFSF4	rs2205960	GG		145	49.2	163	53.1	0.507	Codominant	0.515
		TT		23	7.8	26	8.5		Dominant	0.333
		GT		127	43.0	118	38.4		Recessive	0.763
			G	417	70.7	444	72.3		Allele	0.530
			T	173	29.3	170	27.7			
CTLA4	rs231779	CC		27	9.2	29	9.4	0.588	Codominant	0.912
		TT		137	46.4	147	47.9		Dominant	0.901
		CT		131	44.4	131	42.7		Recessive	0.723
			C	185	31.4	189	30.8		Allele	0.830
			T	405	68.6	425	69.2			
PD1	rs36084323	CC		74	25.1	72	23.5	0.517	Codominant	0.635
		TT		68	23.0	64	20.8		Dominant	0.640
		CT		153	51.9	171	55.7		Recessive	0.514
			C	301	51.0	315	51.3		Allele	0.921
			T	289	49.0	299	48.7			
ICOS	rs6726035	CC		87	29.5	74	24.1	0.959	Codominant	0.059
		TT		62	21.0	89	29.0		Dominant	0.135
		CT		146	49.5	144	46.9		Recessive	**0.024**
			C	320	54.2	292	47.6		Allele	**0.020**
			T	270	45.8	322	52.4			
DNAM1	rs763361	CC		129	43.7	137	44.6	0.621	Codominant	0.905
		TT		31	10.5	29	9.5		Dominant	0.825
		TC		135	45.8	141	45.9		Recessive	0.664
			C	393	66.6	415	67.6		Allele	0.718
			T	197	33.4	199	32.4			
LAG3	rs870849	CC		201	68.1	224	73.0	0.306	Codominant	0.312

Genes	SNPs	Genotype	Allele	Controls		ITP		HWE -p	Model / allele	Uncorrected p value
				Count	%	Count	%			
		TT		6	2.0	8	2.6		Dominant	0.194
		TC		88	29.9	75	24.4		Recessive	0.642
			C	490	83.1	523	85.1		Allele	0.312
			T	100	16.9	91	14.9			

SNP, single nucleotide polymorphism; Uncorrected p value calculated with chi-squared test or Fisher's exact test; HWE-p, p value of Hardy–Weinberg equilibrium; **Bold** highlights statistical significance ($p < 0.05$).

Supplementary Table S2

Association between SNPs and ITP severity

Genes	SNPs	Genotype	Allele	Non-severe ITP		Severe ITP		Model / allele	Uncorrected p value
				Count	%	Count	%		
TIM3	rs10515746	CC		165	97.1	128	93.4	Codominant	0.130
		AA		0	0.0	0	0.0	Dominant	0.130
		CA		5	2.9	9	6.6	Recessive	0.130
			C	335	98.5	265	96.7	Allele	0.134
			A	5	1.5	9	3.3		
CD28	rs1980422	TT		126	74.1	107	78.1	Codominant	0.417
		CC		0	0.0	0	0.0	Dominant	0.417
		CT		44	25.9	30	21.9	Recessive	_
			T	296	87.1	244	89.1	Allele	0.451
			C	44	12.9	30	10.9		
TNFSF4	rs2205960	GG		94	55.3	69	50.4	Codominant	0.422
		TT		16	9.4	10	7.3	Dominant	0.390
		GT		60	35.3	58	42.3	Recessive	0.509
			G	248	72.9	196	71.5	Allele	0.698
			T	92	27.1	78	28.5		
CTLA4	rs231779	CC		14	8.2	15	10.9	Codominant	0.676
		TT		81	47.6	66	48.2	Dominant	0.419
		CT		75	44.2	56	40.9	Recessive	0.921
			C	103	30.3	86	31.4	Allele	0.771
			T	237	69.7	188	68.6		
PD1	rs36084323	CC		48	28.2	24	17.5	Codominant	**0.027**
		TT		28	16.5	36	26.3	Dominant	**0.028**
		CT		94	55.3	77	56.2	Recessive	**0.035**
			C	190	55.9	125	45.6	Allele	**0.011**
			T	150	44.1	149	54.4		
ICOS	rs6726035	CC		41	24.1	33	24.1	Codominant	0.761
		TT		52	30.6	37	27.0	Dominant	0.995
		CT		77	45.3	67	48.9	Recessive	0.492
			C	159	46.8	133	48.5	Allele	0.661
			T	181	53.2	141	51.5		
DNAM1	rs763361	CC		79	46.5	58	42.3	Codominant	0.640
		TT		17	10.0	12	8.8	Dominant	0.339
		TC		74	43.5	67	48.9	Recessive	0.721
			C	232	68.2	183	66.8	Allele	0.703
			T	108	31.8	91	33.2		
LAG3	rs870849	CC		117	68.8	107	78.1	Codominant	0.072

Genes	SNPs	Genotype	Allele	Non-severe ITP		Severe ITP		Model / allele	Uncorrected p value
				Count	%	Count	%		
		TT		7	4.1	1	0.7	Dominant	0.069
		TC		46	27.1	29	21.2	Recessive	0.079
			C	280	82.4	243	88.7	Allele	**0.028**
			T	60	17.6	31	11.3		

SNP, single nucleotide polymorphism; Uncorrected p value calculated with chi-squared test or Fisher's exact test; **Bold** highlights statistical significance ($p < 0.05$).

Supplementary Table S3

Association between SNPs and corticosteroid-sensitivity of ITP

Genes	SNPs	Geno-type	Allele	Corticosteroid-sensitive		Corticosteroid-resistant		Model / allele	Uncorrected p value
				Count	%	Count	%		
TIM3	rs10515746	CC		105	98.1	128	93.4	Codominant	0.119
		AA		0	0.0	0	0.0	Dominant	0.119
		CA		2	1.9	9	6.6	Recessive	0.119
			C	212	99.1	265	96.7	Allele	0.123
			A	2	0.9	9	3.3		
CD28	rs1980422	TT		83	77.6	107	78.1	Codominant	0.921
		CC		0	0.0	0	0.0	Dominant	0.921
		CT		24	22.4	30	21.9	Recessive	_
			T	190	88.8	244	89.1	Allele	0.926
			C	24	11.2	30	10.9		
TNFSF4	rs2205960	GG		55	51.4	70	51.1	Codominant	0.999
		TT		10	9.3	13	9.5	Dominant	0.962
		GT		42	39.3	54	39.4	Recessive	0.970
			G	152	71.0	194	70.8	Allele	0.957
			T	62	29.0	80	29.2		
CTLA4	rs231779	CC		12	11.2	12	8.8	Codominant	0.780
		TT		49	45.8	67	48.9	Dominant	0.523
		CT		46	43.0	58	42.3	Recessive	0.712
			C	70	32.7	82	29.9	Allele	0.510
			T	144	67.3	192	70.1		
PD1	rs36084323	CC		26	24.2	29	21.2	Codominant	0.640
		TT		25	23.4	28	20.4	Dominant	0.658
		CT		56	52.4	80	58.4	Recessive	0.682
			C	108	50.5	138	50.4	Allele	0.982
			T	106	49.5	136	49.6		
ICOS	rs6726035	CC		18	16.8	38	27.7	Codominant	0.067
		TT		36	33.6	32	23.4	Dominant	0.059
		CT		53	49.6	67	48.9	Recessive	0.100
			C	89	41.6	143	52.2	Allele	**0.020**
			T	125	58.4	131	47.8		
DNAM1	rs763361	CC		56	52.3	54	39.4	Codominant	**0.029**
		TT		5	4.7	18	13.1	Dominant	**0.044**
		TC		46	43.0	65	47.5	Recessive	**0.025**
			C	158	73.8	173	63.1	Allele	**0.012**
			T	56	26.2	101	36.9		

Genes	SNPs	Geno-type	Allele	Corticosteroid-sensitive		Corticosteroid-resistant		Model / allele	Uncorre-cted p value
				Count	%	Count	%		
LAG3	rs870849	CC		76	71.0	101	73.7	Codominant	0.756
		TT		4	3.7	3	2.2	Dominant	0.734
		TC		27	25.3	33	24.1	Recessive	0.702
			C	179	83.6	235	85.8	Allele	0.517
			T	35	16.4	39	14.2		

SNP, single nucleotide polymorphism; Uncorrected p value calculated with chi-squared test or Fisher's exact test; **Bold** highlights statistical significance ($p < 0.05$).

Supplementary Table S4

Association between SNPs and ITP Refractoriness

Genes	SNPs	Genotype	Allele	Non-refractory		Refractory		Model / allele	Uncorrected p value
				Count	%	Count	%		
TIM3	rs10515746	CC		269	96.1	24	88.9	Codominant	0.115
		AA		0	0.0	0	0.0	Dominant	0.115
		CA		11	3.9	3	11.1	Recessive	0.115
			C	549	98.0	51	94.4	Allele	0.117
			A	11	2.0	3	5.6		
CD28	rs1980422	TT		215	76.8	18	66.7	Codominant	0.240
		CC		0	0.0	0	0.0	Dominant	0.240
		CT		65	23.2	9	33.3	Recessive	_
			T	495	88.4	45	83.3	Allele	0.275
			C	65	11.6	9	16.7		
TNFSF4	rs2205960	GG		149	53.2	14	51.9	Codominant	0.953
		TT		24	8.6	2	7.4	Dominant	0.892
		GT		107	38.2	11	40.7	Recessive	1.000
			G	405	72.3	39	72.2	Allele	0.988
			T	155	27.7	15	27.8		
CTLA4	rs231779	CC		26	9.3	3	11.1	Codominant	0.913
		TT		134	47.9	13	48.1	Dominant	0.730
		CT		120	42.8	11	40.8	Recessive	0.977
			C	172	30.7	17	31.5	Allele	0.907
			T	388	69.3	37	68.5		
PD1	rs36084323	CC		71	25.4	1	3.7	Codominant	**0.034**
		TT		58	20.7	6	22.2	Dominant	**0.011**
		CT		151	53.9	20	74.1	Recessive	0.854
			C	293	52.3	22	40.7	Allele	0.104
			T	267	47.7	32	59.3		
ICOS	rs6726035	CC		68	24.3	6	22.2	Codominant	0.612
		TT		83	29.6	6	22.2	Dominant	0.811
		CT		129	46.1	15	55.6	Recessive	0.417
			C	265	47.3	27	50.0	Allele	0.707
			T	295	52.7	27	50.0		
DNAM1	rs763361	CC		125	44.6	12	44.4	Codominant	0.913
		TT		26	9.3	3	11.1	Dominant	0.984
		TC		129	46.1	12	44.5	Recessive	0.730
			C	379	67.7	36	66.7	Allele	0.879
			T	181	32.3	18	33.3		
LAG3	rs870849	CC		205	73.2	19	70.4	Codominant	0.830

Genes	SNPs	Genotype	Allele	Non-refractory		Refractory		Model / allele	Uncorrected p value
				Count	%	Count	%		
		TT		8	2.9	0	0.0	Dominant	0.751
		TC		67	23.9	8	29.6	Recessive	1.000
			C	477	85.2	46	85.2	Allele	0.999
			T	83	14.8	8	14.8		

SNP, single nucleotide polymorphism; Uncorrected p value calculated with chi-squared test or Fisher's exact test; **Bold** highlights statistical significance ($p < 0.05$).
